# Vascular Disrupting Agent Drug Classes Differ in Effects on the Cytoskeleton

**DOI:** 10.1371/journal.pone.0040177

**Published:** 2012-07-24

**Authors:** Sujeong Kim, Leonid Peshkin, Timothy J. Mitchison

**Affiliations:** Department of Systems Biology, Harvard Medical School, Boston, Massachusetts, United States of America; Weill Cornell Medical College of Cornell University, United States of America

## Abstract

Vascular disrupting agents (VDAs), anti-cancer drugs that target established tumor blood vessels, fall into two main classes: microtubule targeting drugs, exemplified by combretastatin A4 (CA4), and flavonoids, exemplified by 5,6-dimethylxanthenone-4-acetic acid (DMXAA). Both classes increase permeability of tumor vasculature in mouse models, and DMXAA in particular can cause massive tumor necrosis. The molecular target of CA4 is clearly microtubules. The molecular target(s) of DMXAA remains unclear. It is thought to promote inflammatory signaling in leukocytes, and has been assumed to not target microtubules, though it is not clear from the literature how carefully this assumption has been tested. An earlier flavone analog, flavone acetic acid, was reported to promote mitotic arrest suggesting flavones might possess anti-microtubule activity, and endothelial cells are sensitive to even mild disruption of microtubules. We carefully investigated whether DMXAA directly affects the microtubule or actin cytoskeletons of endothelial cells by comparing effects of CA4 and DMXAA on human umbilical vein endothelial cells (HUVEC) using time-lapse imaging and assays for cytoskeleton integrity. CA4 caused retraction of the cell margin, mitotic arrest and microtubule depolymerization, while DMXAA, up to 500 µM, showed none of these effects. DMXAA also had no effect on pure tubulin nucleation and polymerization, unlike CA4. We conclude that DMXAA exhibits no direct anti-microtubule action and thus cleanly differs from CA4 in its mechanism of action at the molecular level.

## Introduction

Tumor vasculature has been attractive target for chemotherapeutic drug since it is fundamental in tumor growth, progression and metastasis. As cancer cells proliferate, their demand for nutrients and oxygen increases. To recruit new blood vessels to the growing tumor, cancer cells secrete various angiogenic factors [1]. In 1972, Folkman's group showed that tumor cells implanted into the avascular cornea of rabbit eye recruited new blood vessels [2], [3] and hypothesized that blocking angiogenesis with a drug would prevent tumor growth and in some cases cause tumor regression. An angiogenesis-blocking antibody, Avastin, is now widely used in combination with cytotoxic drugs to treat tumors, and other anti-angiogenesis drugs are in different stages of development [4].

Vascular disrupting agents (VDAs) present an alternative way to target tumor blood vessels. Unlike anti-angiogenic drugs, VDAs damage established tumor blood vessels. In rodent cancer models, VDAs cause rapid shutdown of blood flow in established solid tumors in minutes, resulting in massive hemorrhagic necrosis in tumors [5], [6]. Remarkably, vasculature outside of the tumor is not damaged, though the molecular or anatomic basis of this differential sensitivity remains unclear.

Current investigational VDAs can be divided into two major groups, microtubule binding agents and flavonoids. Combretastatin A4 (CA4) is the furthest-developed tubulin binding VDA. It binds to the colchicine binding site in tubulin and depolymerizes microtubules, but is less toxic than colchicine [7]. The first anti-cancer flavonoid, FAA, was originally identified by the Developmental Therapeutic Program, Division of Cancer Treatment, NCI as an antitumor agent in mice [8]. FAA had little activity in humans. Baguley and colleagues identified DMXAA as a more potent derivative [9]. Currently, CA4 and related compounds are in phase I/II/III clinical trials DMXAA is in phase III trials in both cases for treatment of intractable cancers in combination with standard chemotherapy [10], [11], [12]. So far, DMXAA has not exhibited the high anti-tumor efficacy in humans that was seen in mouse models, but it remains a conceptually exciting drug.

Despite promising results in rodent models, and some evidence of clinical efficacy, the molecular, cellular and tissue mechanisms of VDAs remain poorly understood. This lack of mechanistic understanding has hindered clinical development, making it hard to develop predictive or response biomarkers, or in the case of DMXAA, more potent derivatives. CA4 clearly targets microtubules and reorganizes actin cytoskeleton resulting membrane blebbing [13], [14], [15], but how this leads to vascular permeabilization, and why this effect is tumor-selective, remain unclear. DMXAA is known to stimulate white blood cells to secrete various cytokines in mouse by an unknown pathway that requires the kinase TBK1 (TANK binding kinase 1) activity [16]. Tumor necrosis factor-alpha (TNF-alpha) secretion seems important for DMXAA action *in vivo*, since its anti-tumor activity was impaired, though not completely lost, in TNF-alpha receptor knock-out mice [17], [18]. Therefore, the major action of DMXAA on endothelial hyper-permeability appears to be indirect, via leukocyte-mediated signaling. However, DMXAA has been reported to directly trigger signaling changes and apoptosis in endothelial cells [19] and endothelial barrier function is exquisitely sensitive to microtubule disruption [20], [21], [22]. DMXAA is not thought to target microtubules, but to our knowledge this point has not been critically addressed in the literature. FAA was reported to cause G2/M arrest at high concentrations [23], suggesting a potential for anti-microtubule action by the flavonoid class, and tubulin is known to bind structurally diverse aromatic molecules. In this study, we critically evaluated whether DMXAA has anti-microtubule activity in endothelial cells. Our results are negative, and thus support the widespread assumption that the two VDA classes differ in mechanism, but we feel this result is nevertheless a useful contribution to the VDA literature.

## Results

**Figure 1:**
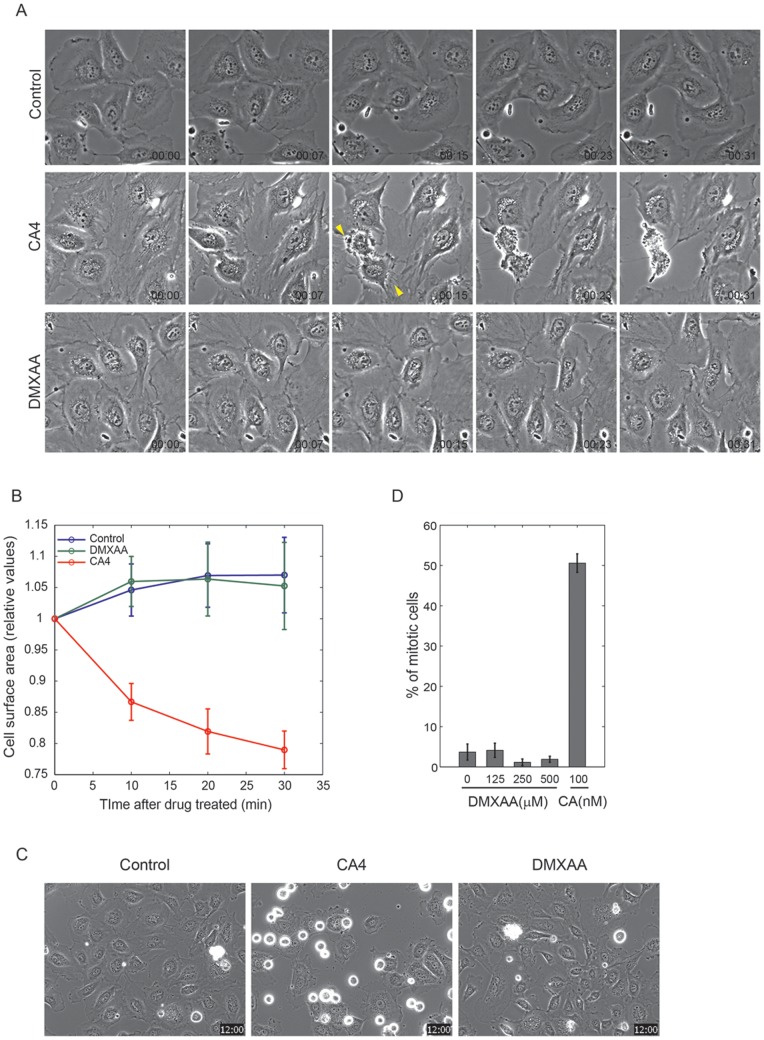
DMXAA does not induce any morphological changes in HUVEC cells whereas CA4 treated cells immediately contract. (A) Images were collected from phase contrast time-lapse movies at indicated times. Original time-lapse imaging was taken every 30 sec for an hour in the presence of 500 µM of DMXAA, 100 nM of CA4 or without drug (control). Elapsed time indicated in hours:minutes. (B) Cell edges were drawn to measure the number of pixels within the cell edges. Individual cell areas were summed to measure total cell surface area at each time point. The total cell area for each time point was normalized by time point 0. At each condition, Cell surface areas were averaged from at least 3 different stage positions. Error bars were calculated as the standard deviation from the results of 3 independent experiments. (C) Images were collected from phase contrast time-lapse movies at 12 hours after drug treatment. Asynchronously grown cells were treated with 500 µM of DMXAA, 100 nM of CA4 or without drug (control). (D) From the time-lapse image, mitotic cells were counted after 12 hours of drug treatment. At least 200 cells from 3 different stage positions were counted at each condition. Error bars were calculated as the standard deviation from the results of 3 independent experiments.

To compare the effects of CA4 and DMXAA on endothelial cells we performed time-lapse imaging of HUVEC cells before drug, and in drug for 30 min (Fig. 1). Live cell microscopy can sensitively report effects on cytoskeleton, adhesion, proliferation and the signaling pathways that control them. CA4 caused rapid contraction and loss of cell-cell interaction starting within minutes of drug addition (Fig. 1A middle panel) as previously described [13]. This response was evident from retraction of cell margins and formation of thin retraction fibers. Retraction fibers terminated at the cell body in characteristic phase-dense structures (yellow arrows), similar to those previously characterized in cells rounding up for mitosis [24]. DMXAA and none treated control HUVEC cells showed no signs of retraction (Fig. 1A lower panel and upper panel). These differential effects on retraction were quantified by image analysis which revealed time-dependent retraction in CA4 but not DMXAA treated cells (Fig. 1B). Total cell surface area was decreased about 20% in CA treated cells after 30 min whereas control and 500 µM of DMXAA had no effect (Fig. 1B).

Microtubule-targeting drugs disrupt mitotic spindle assembly, leading to activation of the spindle assembly checkpoint and mitotic arrest [25]. Thus mitotic index and mitosis duration are sensitive markers of microtubule disruption. Using phase contrast microscopy, mitotic indices were quantified as previously described [26]. As expected, CA4 treated HUVEC cells gradually accumulated in mitotic arrest, leading to a mitotic index of up to 50% at 12 hours of drug treatment (Fig. 1C and D). They remained in mitotic arrest for prolonged period, and mostly died after 12 to 16 hours of arrest (Data not shown), presumably by activation of the intrinsic apoptosis pathway [27]. DMXAA caused no increase in mitotic index and it was about the same compared to vehicle control. At high concentrations of DMXAA, 250 µM and 500 µM, the fraction of mitotic cells were 1.6% and 1.5% respectively, which is slightly lower than the control cells without drug (3.7%), suggesting a mild inhibition of cell cycle progression.

**Figure 2:**
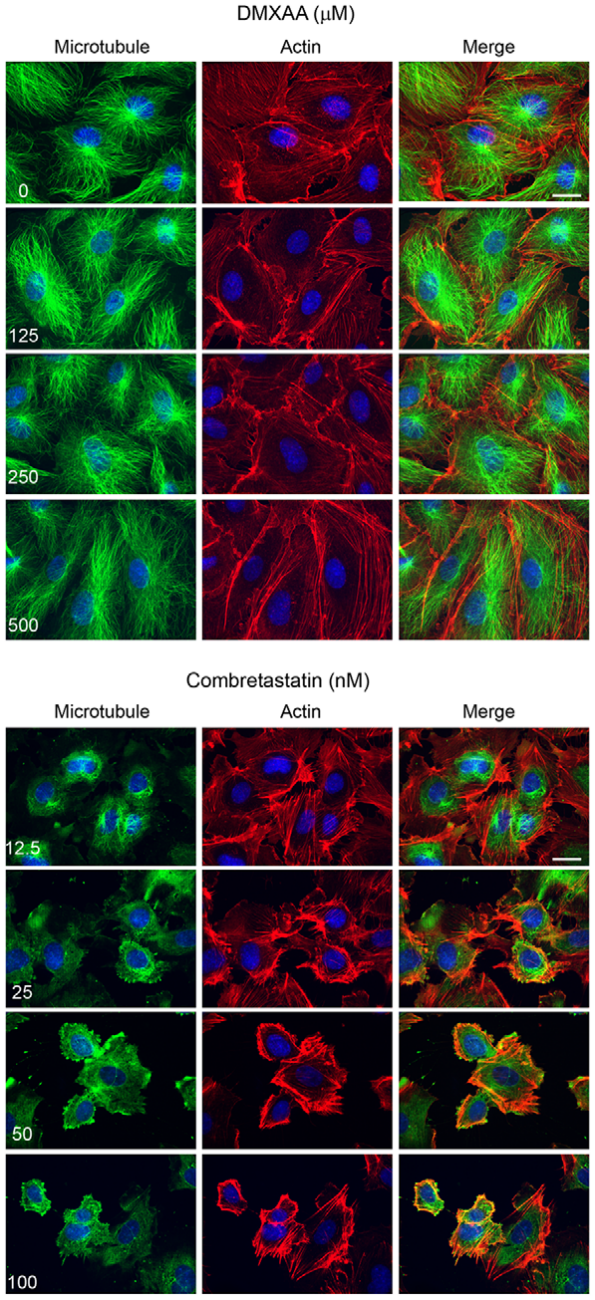
DMXAA did not disturb microtubule or actin structure. Cells were treated with serial dilutions of DMXAA for 30 min and CA4 for 10 min. Immuno-staining was performed using anti-DM1alpha and rhodamine-phalloidine to visualize microtubule and actin respectively. Microtubules are shown in green and actin in red. Nuclei were stained using DAPI (blue). The white bar indicated 20 µm.

To test directly if DMXAA affects the microtubule or actin cytoskeleton in endothelial cells, we imaged microtubule and actin in fixed, drug-treated HUVEC cells by spinning disk microscopy (Fig. 2). In control cells, microtubule structures radiated out from the centrosomes and thin actin bundles were visible throughout the cell (Fig. 2 upper panel, top row of images). CA4 induced dose-dependent microtubule disruption, starting at 12.5 nM. In cells treated with this minimal concentration, the remaining microtubules lost their radial structure and were tangled up and around the centrosome (Fig. 2 lower panel). Actin bundles appeared thicker and brighter. At 100 nM CA4 microtubules were completely depolymerized and actin bundles appeared even more prominent. No changes in microtubules were noted following DMXAA treatment up to 500 µM (figure 2, upper panel).

**Figure 3:**
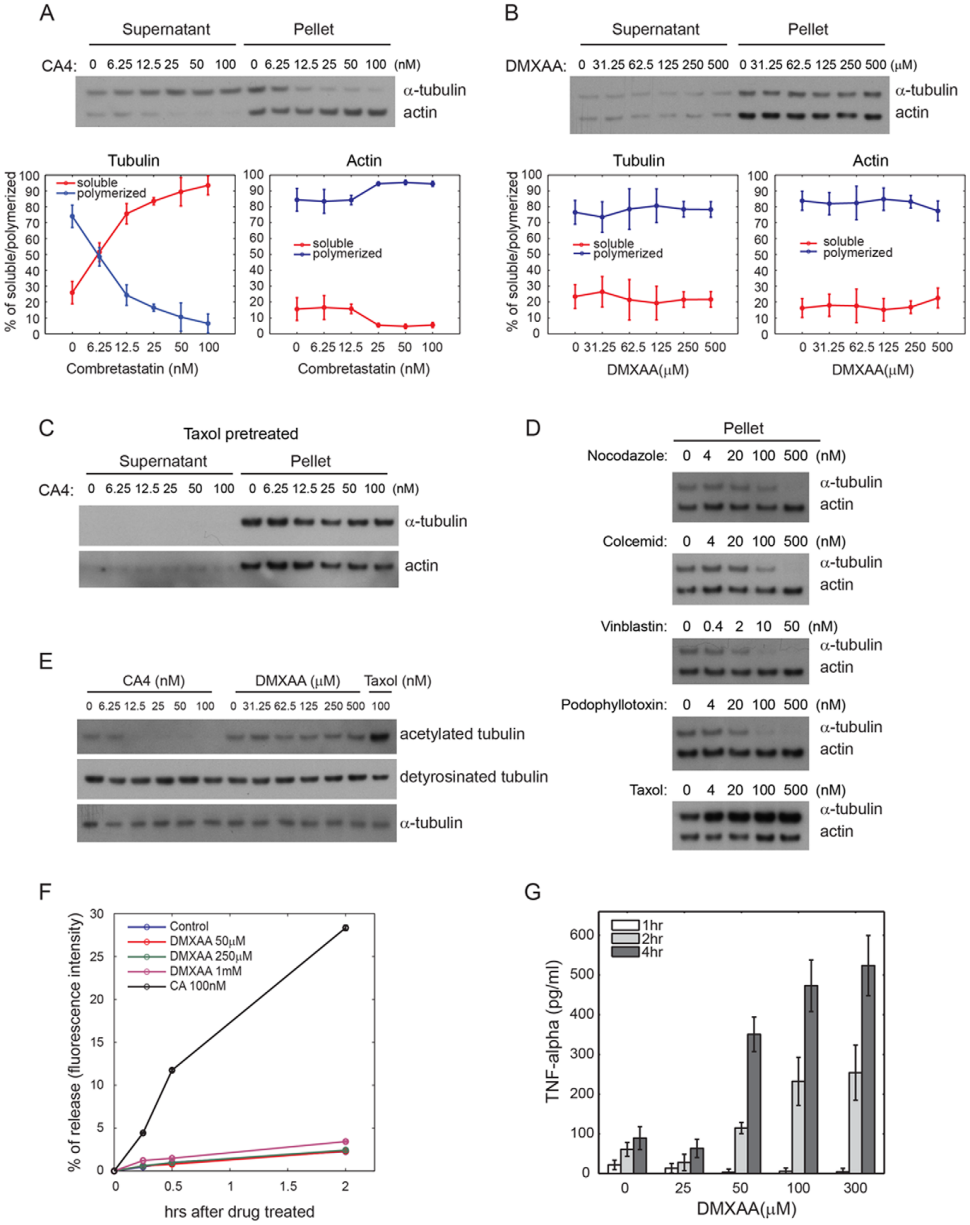
Quantitative analysis of the polymerization state of microtubules and actin in drug treated cells. (A) Soluble tubulin extraction assays were performed after treatment with serial dilutions of CA4 for 10 min. Tubulin and actin monomers were extracted from supernatant but polymerized tubulin and actin remained in the pellets. Each supernatant and pellet was subject to Western blotting to measure protein levels of tubulin and actin. The ratio of soluble vs. polymerized tubulin and actin are plotted in the lower panel. Error bars were calculated as the standard deviation from the results of 4 independent experiments. (B) DMXAA was treated in serial dilution for 30 min. The same experiment was performed as in (A). (C) Cells were pretreated with 300 nM of Taxol for 30 min before CA4 treatment. And soluble tubulin and actin extraction assay was performed as (A and B). (D) Various microtubule targeting drugs were treated as indicated time for 15 min and followed by soluble tubulin and actin extraction assay. And only pellets in each condition were subjected to detect polymerized actin or tubulin. (E) Cells were treated with CA4, DMXAA or Taxol as indicated concentrations and total cell lysates were analyzed by western blotting. (F) HUVEC monolayer permeability assay. Cells were grown on 3 µm pore membrane inserts for 2 days and DTAF labeled 3 k dextran (50 µg/ml) was added in insert with or without drug (control). Fluorescence intensity of the lower chamber was measured to analyze the permeability of the monolayer. The percent of released dextran was plotted as the fluorescence intensity of the lower chamber relative to the fluorescence intensity of the upper chamber intensity. (G) TNF-alpha ELISA assay. Media were collected after DMXAA treatment for various time and concentration for TNF-alpha ELISA assay. Error bars were calculated as the standard deviation from the results of 5 independent experiments.

The lack of a direct effect of DMXAA on the cytoskeleton of endothelial cells we observed by immunofluorescence appeared to contradict literature reports [28], [29]. To validate the activity of the batch of commercial DMXAA we used, we tested its effect on TNF-alpha secretion by the mouse macrophage like cell line Raw264.7 (Fig. 3G). Our batch of DMXAA induced TNF-alpha secretion with a time and dose dependence similar to published data [30]. Thus our DMXAA batch has the expected activity on macrophages, and its lack of effects on the endothelial cell cytoskeleton is likely to be a reliable negative result.

To obtain more quantitative data on possible cytoskeleton effects of DMXAA, we assayed the fraction of tubulin and actin in the monomer and polymer pools with a cell permeabilization assay (Fig. 3). In brief, HUVEC cells were treated with DMXAA at different concentrations for 30 min, or CA4 for 10 min. We avoided longer exposure to CA4 since retracted cells tend to lose substrate attachment during permeabilization. Drug-treated cells were permeabilized using a non-ionic detergent (triton X-100) in a microtubule and F-actin stabilizing buffer for 2 min. Unpolymerized protein was released into the supernatant and polymer remained associated with the substrate. Tubulin and actin in both fractions were quantified by Western blotting. Band intensities were analyzed using ImageJ and percent of soluble or polymerized tubulin or actin was plotted over total tubulin or actin (soluble plus polymerized) (fig. 3A, lower graphs). Without drug, approximately 75% of tubulin was polymerized and 25% was soluble in control cells (without drug). CA4 caused dose-dependent microtubule depolymerization with an EC50 of ∼5–10 nM (fig. 3A). DMXAA, in contrast, had no effect on the fraction of polymerized tubulin (fig. 3B). The fraction of actin in polymerized form reproducibly increased following CA4 treatment, consistent with the imaging data (fig. 3A, lower graph). The EC50 value was slightly higher for actin polymerization than for tubulin depolymerization, suggesting most microtubules must be depolymerized for the actin cytoskeleton to respond.

To test whether microtubule depolymerization is necessary for CA4 to induce actin polymerization, we pretreated taxol to prevent microtubule depolymerization (fig. 3 C). Taxol pretreatment completely blocked microtubule disruption by CA4. Interestingly, the response of actin to CA4 was decreased by taxol pretreatment suggesting microtubule disruption is required for actin polymerization by CA4. Then we checked other microtubule targeting drugs to check whether this effect was specific to CA4 (Fig. 3D). We compared nocodazole, colcemid, vinblastine and podophyllotoxin to disrupt microtubules and taxol to stabilized microtubules. All the depolymerizer decreased the amount of insoluble tubulin as expected. However, we the increase in actin polymerization was unique to CA4 compared to any other microtubule disrupting drug. Only the microtubule stabilizing drug taxol clearly increased polymerized actin. These data suggested that CA4 has an effect on microtubules that differs, perhaps in subtle ways, from other depolymerizers. For example, it might partially stabilize microtubules at threshold concentrations. Its induction of actin polymerization was presumably indirect via the Rho GTPase pathway as reported [13]. On the other hand, DMXAA caused a slight decrease in actin polymerization at the highest concentrations, so its effects on the endothelial cell cytoskeleton were, if anything, opposite to those of CA4 (Fig. 3B). Lastly, we checked post translational modification of tubulin such as acetylation and detyrosination (fig. 3E). Consistent with permeabilization assay, there were no changes in acetylated or detyrosinated tubulin levels in DMXAA treated cells whereas acetylation clearly decreased in CA4 treated cells. Detyrosinated tubulin levels were not changed in any conditions.

Next, we performed an endothelial barrier permeability assay. HUVEC cells were seeded on 3 µm pore membrane inserts and grown to form confluent monolayers. DMXAA or CA4 were added, along with fluorescently labeled 3 k dextran, to the upper chamber and medium was collected from the lower chamber at each time point. Data are reported as lower chamber fluorescence intensity was divided by upper chamber intensity (Fig. 3F). CA4 treatment strongly increased dextran barrier crossing, while DMXAA had no effect compared to control treatment.

**Figure 4:**
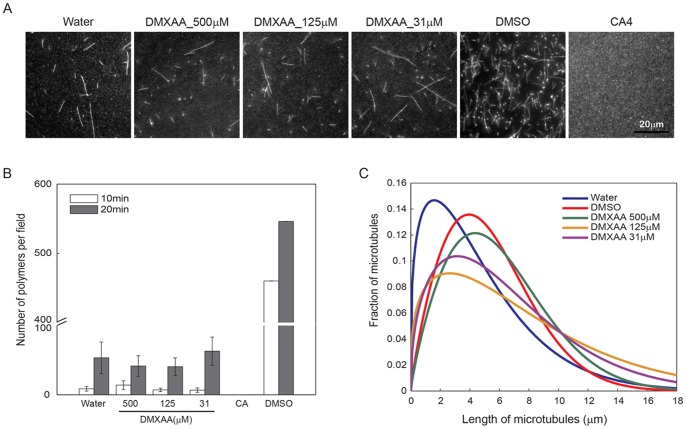
Comparison of the effects of CA4 and DMXAA on tubulin polymerization. Polymerized tubulin was visible by fluorescence microscopy as string like shapes. Various concentrations of DMXAA and its vehicle control (water) showed similar tubulin polymerization rates. DMSO was used as a positive control. The white scale bar indicates 20 µm. All images were taken after 20 min polymerization. (B) The number of polymers per field were counted using ImageJ. Three to ten different random stage positions were counted to measure number of polymers for each condition, except for DMSO treated were one stage position was counted. Error bars were calculated as the standard deviation from the results of 3 independent experiments. (C) The Weibull-parametrized distribution of microtubule length compared across conditions in 20 min polymerization samples. The lengths were measured using ImageJ and fit to a Weibull probability density function for each condition.

Finally, we performed *in vitro* polymerization assays with pure tubulin in the presence of drugs to further test the possibility that DMXAA directly destabilizes microtubule (Fig. 4). In brief, pure tubulin derived from bovine brain, containing a small fraction of subunits covalently labeled with a fluorescent dye, was mixed with or without drug in GTP containing polymerization buffer. Samples were incubated at 37°C for 10 to 20 min to polymerize microtubule, then fixed and imaged. This assay scores for effects on both nucleation and elongation of microtubules, and the tubulin concentration was adjusted to the minimal value needed to observe polymerization, to maximize the sensitivity of the assay to potential inhibitors. As shown in fig. 4A, CA4 completely blocked microtubule polymerization, while DMXAA did not show any significant inhibition. It is important to compare the effect of each drug to that of its vehicle, which was water for DMXAA (dissolved as its sodium salt) and DMSO for CA4, since DMSO alone promotes microtubule polymerization. The total number of microtubules and their length were manually quantified using ImageJ (fig. 4 B and C). In order to represent the distribution of the microtubule lengths obtained from many dozens of measurements, we fit a two-parameter Weibull probability density function using MATLAB implementation. DMXAA has little effect on total microtubule number per field (Fig. 4B), but consistently caused a modest shift in the length distribution towards a higher fraction of long microtubules (Fig. 4C). Overall the effect of DMXAA on pure tubulin polymerization was very mild, and if anything there was a slight increase in polymerization, unlike the complete inhibition caused by CA4.

## Discussion

In this study, we directly compared the effect of representative agents from the two main classes of VDA drugs on endothelial cells. Such a head-to-head, quantitative comparison is, to our knowledge, missing from the literature. We felt it was important because of some hints in the literature that flavonoid-class drugs cause mitotic arrest or disrupt the endothelial cell cytoskeleton. Using time-lapse microscopy, immuno-staining, soluble tubulin extraction assays and endothelial cell permeability assays we confirmed that CA4 depolymerizes microtubules at low concentrations, which leads to an increased fraction of polymerized actin, cell retraction, loss of endothelial barrier function and eventual mitotic arrest of HUVECs. DMXAA had none of these effects, confirming the widely held view that it acts in an entirely different way. The microtubule polymerization assay (fig. 4) showed that there might be mild positive effects of DMXAA on tubulin polymerization as overall tubulin polymer sizes were longer in the presence of DMXAA. This result suggests that DMXAA might stabilize microtubules. However, as shown in fig. 1, there is no mitotic arrest in DMXAA treated cells, which is a sensitive assay for perturbation of microtubule dynamic. Thus it is unclear that the mild effect of DMXAA on pure tubulin is physiologically significant. In any case it goes, if anything, in the opposite direction compared to CA4.

Our data leave open the question of whether DMXAA has any action on endothelial cells that are related to its VDA action *in vivo*. Baguley's group showed that high concentration of DMXAA (400 µg/ml) increased endothelial cell death by an unknown pathway in the murine endothelial cell, HECPP [19]. Also while we revised this manuscript, Lou group reported that DMXAA induced rapid morphological changes and actin reorganization via p38 pathway in HUVEC cells [29]. At the moment, we do not have explanation why we got opposite results with Lou group. We know our DMXAA is fully active, based on its effects on TNF-alpha secretion from Raw264.7 cells (Fig. 3G). Although we could not find how Lou group dissolved DMXAA in their paper, this could be important point. The only effects of DMXAA that we observed on endothelial cells were a mild decrease in the fraction of actin in polymer (fig. 3) and a mild decrease in HUVEC proliferation rate, manifest by lower mitotic index and also in 24 hr growth assays (not shown). Both effects were only seen at high DMXAA concentrations (250–500 µM). We noted similar or stronger growth inhibition in cancer cell lines (HeLa, A549) and Raw264.7 cells by DMXAA (data not shown), so these effects are probably not endothelium specific. We definitely did not observe a strong increase in apoptosis by time-lapse imaging (not shown). Whether these relatively mild effects at high DMXAA concentration represent specific effects of the drug that are relevant to VDA activity is unclear. In terms of effect on cell physiology that seem much less impressive than the cytokine secretion promoting activity of DMXAA on leukocytes.

Taken together, or results suggest that DMXAA had little or no effect on the morphology, cytoskeleton or barrier function of cultured endothelial cells. This supports the standard literature assumption that the two classes of VDA work by fundamentally different mechanisms, and provides the first quantitative evidence (to our knowledge) that DMXAA lacks anti-microtubule effects. We did detect some mild effects of DMXAA in our assays, but only at the highest concentrations. It exhibited mild anti-proliferative activity on HUVECs as assayed by decreased mitotic index, mild decrease in actin polymer on HUVECs, and mild stabilization of microtubules in a pure tubulin assay. We doubt that the *in vitro* effect on microtubules is physiologically significant given the lack of mitotic arrest and the lack of evidence for microtubule stabilization *in vivo*.

In conclusion, we our data bring strong new evidence in support of the widely-held literature assumption that DMXAA does not act via microtubule destabilization like CA4. So how and which cytokines regulate endothelial hyper-permeability would be an important question to be addressed in the future.

## Materials and Methods

### Cell culture and reagents

Human umbilical vein endothelial cells (HUVECs) were purchased from LONZA (Walkersville, MD, USA). Cells were maintained using EGM-2 Bullet kit (LONZA, Walkersville, MD, USA) in a humidified incubator (37°C, 5% CO_2_). For experiments, cells were seeded on fibronectin (50 µg/ml) (Sigma-Aldrich, St Louis, MO, USA) coated dishes. Raw264.7 cells were purchased from ATCC (Manassas, VA, USA). Cells were grown in DMEM medium (Cellgro Mediatech, Manassas, VA, USA) supplemented with 10% heat inactivated FBS (Gibco, Grand Island, NY, US). DMXAA was purchased from Wuhan Sunrise Technology (Wujiashan, Wuhan, China) and CA4 was from Sigma (Sigma, St Louis, MO, USA). DMXAA was dissolved in equimolar sodium bicarbonate buffer overnight and lyophilized to make DMXAA sodium salt. For experiments, DMXAA sodium salt was dissolved in water at 50 mM stock concentration and diluted using culture medium to the desired concentration. CA4 was dissolved in DMSO.

### Time-lapse imaging and image analysis

Cells were grown on fibronectin coated 24 well glass bottom dishes (No. 1.5) (MatTek Corporation, Ashland, MA, US). Plates were mounted on a Prior Proscan II motorized stage in a custom-built microscope incubator (HMS machine shop) maintained at 37°C and 5% CO2. A layer of mineral oil on top of the cell culture media was used to prevent evaporation. All images were collected with a TE2000E motorized inverted microscope (Nikon Instruments, Melville, NY, US) using phase contrast. Images were acquired with a Hamamatsu ORCA ER cooled CCD camera (Hamamatsu Photonics, Bridgewater, NJ) controlled with MetaMorph 7 software (Molecular Devices, Inc., Sunnyvale, CA, US). For time-lapse experiments, images were collected every 30 seconds for cell surface area analysis or 5 minutes for mitotic index analysis, using an exposure time of 50 ms and 2×2 binning. For cell surface analysis, each individual cell was outlined to measure number of pixels within the cell using image J and cell surface areas were calculated by sum of number of pixels of all cells at each time points. Relative cell surface areas against time 0 were plotted.

### Immunofluorescence and confocal miscrscopy

Cells were grown on No. 1.5 coverslips for one day and treated with DMXAA for 30 min or CA4 for 10 min and fixed using 4% formaldehyde (Polysciences, Inc., Warrington, PA, US) in CBS buffer (10 mM MES (pH 6.1), 138 mM KCl, 3 mM Mgcl2, 2 mM EGTA). Fixed cells were permeablized using 0.5% of tritonX-100 (Sigma, St Louis, MO, US) in TBS for 10 min and then blocked and incubated with AbDil (0.1% tristonX-100 and 2% BSA in TBS). Microtubules and actin were visualized using FITC tagged DM 1-alpha antibody (Sigma, St Louis, MO, US) and phalloidin-TRITC (Sigma, St Louis, MO, US) respectively. Nuclei were stained using DAPI (Sigma, St Louis, MO, US). Fluorescence images were collected by Nikon TE2000U inverted microscope with Nikon 1.4 NA DIC optics, 60X oil immersion objective (Nikon, Meville, NY). Confocal images were obtained using, Yokogawa CSU-10 spinning disk confocal head (Yokogawa Corporation of America, Newnan, GA, USA) with Sutter emission filter wheel. Images were acquired with Hamamatsu ORCA-AG cooled CCD camera (Hamamatsu, Hamamatsu, Japan) controlled with MetaMorph 7 software (Universal Imaging, Molecular Devices, Sunnyvale, CA).

### Soluble tubulin and actin extraction assay

Cells were incubated with tubulin extraction buffer (60 mM PIPES, 25 mM HEPES, 10 mM EGTA, 2 mM MgCl2, 0.5% tritonX-100 and 10 µg/ml of taxol) containing protease inhibitor (Roche Diagnostics, Indianapolis, IN, USA) for 2 min. Supernatant was collected for the soluble fraction and the remaining adherent cells were harvested for the polymerized fraction. Both fractions were lysed using Western blot sample buffer (Invitrogen, Grand Island, NY, US) and an equal volume of each fraction was subjected to Western blotting.

### Western blot

Cells were lysed using 1X protein sample buffer (Invitrogen, Grand Island, NY, US)and. separated by SDS PAGE. Protein was transferred to nitrocellulose membrane (pore size 0.2 µm). Both anti-alpha tubulin and anti-beta actin antibodies were purchased from Sigma (St Louis, MO, USA).

### Endothelial permeability assay

HUVECs were plated onto fibronectin coated BD BioCoat 3 µm pore inserts (BD Biosciences, Franklin Lakes, NJ, US) and grown until confluent. Drugs were added to the upper chamber with DTAF tagged 3 k dextran (60 µg/ml) (Sigma, St Louis, MO, USA). At each time point, the lower chamber media was collected and analyzed in a Victor multilabel plate reader (PerkinElmer, Waltham, MA, US).

### Tubulin polymerization assay

Tubulin polymerization assays were performed as previously described [31]. Briefly, labeled and unlabeled tubulin were mixed at a ratio of 5∶1 to a final concentration of 30 µM in 2 mM GTP containing BRB80 buffer (80 mM K PIPES (pH 6.8), 1 mM MgCl2, 1 mM EGTA). This mix was incubated at 37°C and 2 µl aliquots of each condition were taken at 10 min and 20 min. Aliquots were diluted into fixation buffer (60% glycerol, 0.1% glutaraldehyde in BRB80 buffer) and gently inverted 5 times. This mixture was placed on a glass slide, covered with 22 by 22 mm coverslip and observed with a fluorescence microscope. The number and the lengths of polymerized microtubules were analyzed using ImageJ.

### TNF-alpha ELISA assay

Raw264.7 was seeded on 96 well plate at a density of 2×10^5^ per ml. Cells were treated with DMXAA for various time. And media were collected for TNF-alpha ELISA assay. TNF-alpha ELISA assay (Biolegend, San Diego, CA, USA) was performed as manufacturer's recommendation.
